# Application of social media in the environment and health professional community

**DOI:** 10.1186/1476-069X-11-S1-S16

**Published:** 2012-06-28

**Authors:** Sonja Grossberndt, Peter van den Hazel, Alena Bartonova

**Affiliations:** 1 NILU – Norwegian Institute for Air Research, Instituttveien 18, 2027 Kjeller, Norway; 2 Public Health Services Gelderland-Midden, Eusebiusbuitensingel 43, 6828 HZ Arnhem, The Netherlands

## Abstract

The purpose of the EU FP6 funded coordination action HENVINET was to create a permanent network of environment and health professionals. The main outcome is a networking portal (http://www.henvinet.eu), based on the concepts of social media to support communication between professional stakeholders in the environment and health fields. Its aim is to enable sharing of relevant information in an innovative and interactive manner to eventually support policy making. A social networking tool is not necessarily a typical platform for communication in the professional context, or between scientists and decision-makers. The aim of this paper is to look upon the use of social media in relevant professional communities in the light of the HENVINET experience, and to reflect on the acceptance and usefulness of such a new approach.

The portal was designed over the course of HENVINET through intensive interactions by a multi-disciplinary group, involving environmental as well as health scientists, but with only limited access to decision-makers’ opinions. After the social networking portal was launched, a recruitment campaign was run during the last six months of the project, taking every opportunity to present the portal and to get feedback from users. This feedback was used to improve the functionalities of the tool.

Additionally, a feedback session was organized at the final event of the project, attended by over 50 professionals, about half of whom participated from the beginning in the entire HENVINET project. We have also compared the HENVINET portal with similar tools employed by other related communities, and made a literature-based survey on the use of social media for scientific communication.

At the end of the project, the portal had more than 300 members with registered professional profile, over 10 topics and 15 discussion groups. The HENVINET consortium members were the most active group of users. The quality of the portal content was considered more important than having a large amount of information. To maintain the content, the majority of the participants declared their willingness to use their time, stating however that dedicated content providers would be also necessary.

In theory, professionals see the value of such a tool, and are willing to contribute. Only time will tell if the tool is viable in the long run.

## Introduction

To protect the health of the general population, policies need to integrate both environmental and health (E&H) strategies. Good communication strategies are indispensable, not only within the E&H community, but also between the two fields and additional stakeholders, last but not least decision-makers [1-3].

The traditional evidence-based culture sees scientific evidence as a major source of information for E&H policy-makers to use within their decision-making process. During the last decades, however, traditional evidence-based science (‘Mode 1’) has been losing its autonomy and is now gradually complemented by a *‘new paradigm of knowledge production* (*‘Mode 2’*)*’* that is *‘socially distributed*, *application-oriented*, *trans-disciplinary*, *and subject to multiple accountabilities’ *[4]. At the same time policy people have to deal to an increasing degree with issues characterized by complexity and inter-linkages: decisions are urgent, stakes are high and diverse, values are in dispute, uncertainty and ignorance involved are high, and trust is fragile [5,6]. Against this background, evidence-based E&H decision-making needs to be understood as a process with multiple sources of information and research evidence. The scientific sources may include not only research results, but also – depending on the particular background – internal program evaluations, best practices, or experiences from other programs or initiatives [7]. Political decisions are also influenced by the weight of political, social/cultural and economical factors [8]. Different types of policies and the complexity of the policy process require different types of relevant evidence [9]. Complexity and uncertainty are playing an even bigger role in the context of decision-making on an international level [10].

In the context of information uptake and dissemination, the role of social media has become more and more important, transforming media monologues into dialogues [11]. Social media, defined by *Kaplan & Haenlein* as *‘a group of Internet-based applications that build on the ideological and technological foundations of Web 2.0*, *and that allow the creation and exchange of User Generated Content’*, have during the last two decades taken a solid position within global communication [12]. To an increasing degree the general public, and even decision-makers, tend to use electronic communication channels to become exposed to current scientific research as well as further information of interest, especially via the internet [13]. This leads to the question: **Are social media also suitable for dissemination and communication between different stakeholders in E&H?** This paper will provide insight into the use of social media approaches in this context by reference to results of the EU project HENVINET.

## The HENVINET approach

The EU FP6 project HENVINET (Health and ENVIronment NETwork) was designed to support the EU policy making process towards an integrated approach on environment and health (E&H). By focussing on the four priority health issues defined by the EU Environment and Health Action Plan (EHAP) 2004-2010 (Asthma and Allergies, Cancer, Neurodevelopmental Disorders, Endocrine Disrupting Effects) [14], HENVINET aimed to provide a structured information overview. One of the main project outcomes was the development of an interactive networking portal (http://www.henvinet.eu), based on social media principles, to bring together E&H experts from different backgrounds to improve communication and collaboration in order to establish informed and substantiated political decision-making. The objective of the portal is *‘to provide a platform to facilitate networking for professionals*, (*...*) *facilitate networking through providing a professional virtual meeting place to make new contacts*, *maintain contacts*, *discuss issues*, *and share information for the purpose of better informed policy making’ *[15]. It is for this reason that a social media based portal was designed to facilitate the communication process between science and policy. The general concept is based on Facebook©-type principles, but geared towards professionals in the E&H community. Features accessible for all users are: Groups, Forum, Events, Tools, and Documents. Additionally, registered users have access to a Profile page, the Members page and the option to participate in groups and forum discussions. They are subscribed automatically to the newsletter, which contains news and recent site activities. As a registered user it is also possible to search the members’ area for experts in one’s field(s) of interest for direct contact. The portal contains moreover a number of tools and outcomes of the HENVINET project, such as a searchable Meta Data Base (MDB) of Decision Support Tools (DSTs), Causal Chain Diagrams with evaluation, policy briefs and links to the project web sites [15]. Upcoming events can be announced at the designated calendar section. As of March 01, 2011, the portal had 350 members from various stakeholder groups such as Agency, Authority, Consulting, Education, Industry, NGO, Research, and Other. 1,045 visits from 46 countries worldwide have been counted so far. 65% of the visitors find the portal directly, 9% get redirected via referring sites, and 26% via search engines [16].

## Voting session ‘Communication Strategies for Environment and Health’

At the end of its four years’ course, the HENVINET project organised the final event ‘Approaching Complexities in Environment and Health’ from 14-15 April 2010 in Brussels, Belgium. The main aim of the conference was to provide a platform for sharing methods and experiences in E&H as well as for discussing expectations that the E&H communities have towards each other. The session ‘Communication Strategies for Environment and Health’ was used to receive feedback from the participants by means of an interactive voting system on the following areas:

1. Stakeholder analysis

2. Stakeholders’ needs

3. Stakeholder involvement into the HENVINET portal activities

4. Science-policy interface

5. Decision Support Tools (DSTs)

The answers from the voting session provided valuable information about the use of the HENVINET networking portal and delivered suggestions for further development of this kind of social media for E&H.

A Personal Response System (PRS) or voting system is a form of technology that permits an audience to reply to questions or statements individually. The PRS is very easy to use and offers a method of active engagement. Some research has found that it has a very significant effect on students’ performance in lectures, stimulating their interest and concentration [17] and it creates greater engagement and broader participation [18]. Furthermore, it increases the audience’s enjoyment of lectures and it has proved to be an excellent method of encouraging active learning. At the final HENVINET meeting, 53 conference attendees participated in the interactive voting session ‘Communication Strategies for Environment and Health’. They received 11 questions with multiple answer options but could give only one vote per question by means of a Personal Response System (PRS). In order to give a vote, each participant was given a keypad which transmitted their answers automatically on radio frequency to a receiver. The receiver was connected to a special plug-in for Microsoft Power Point© which made it possible to display the results immediately on a big screen, visible for the entire audience. Each voting question was followed by a short discussion round where the participants had the opportunity to comment on the results.

### Stakeholder analysis

**Figure 1:**
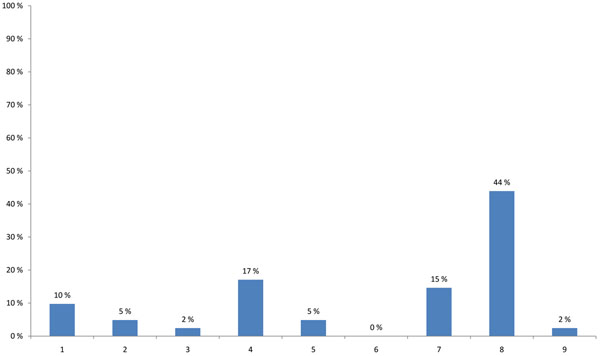
**Participant’s background (multiple choice)** 1 - Developing policy/legislation related to Environment and Health 2 - Applying policy/legislation related to Environment and Health 3 - Addressing stakeholder interests (Industry, NGO, …) 4 - Providing public information on Environment and Health 5 - Medical practice 6 - Consulting 7 - Developing risk assessment / decision support activities 8 - Research 9 - Other

**Figure 2:**
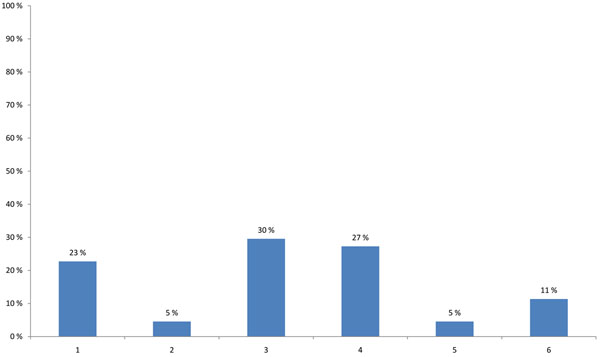
**Most frequent way of communication (multiple choice)** 1 - I write reports which are sent to the person or organisation who gave me the job. 2 - I call regularly with journalists to provide them with information or produce press releases. 3 - I present my work results in workshops or conferences. 4 - I write mainly articles about my results. 5 - I do not communicate myself. 6 - Other ways of communication.

The HENVINET final event brought together participants with varying professional backgrounds and expertise. The highest proportion of participants were researchers (44%), followed by stakeholders developing decision support activities and policies (25%) or providing public information on E&H (17%) (Figure 1). When asked about the modes of communication used, 57% of the participants stated that they use the traditional dissemination routes like conferences, meetings and journals and 23% wrote reports for the organisations/persons they are employed with. Contact with journalists was named only in 5%, while 11% used other ways of communication and 5% did not communicate their results (Figure 2).

### Stakeholders’ needs

**Figure 3:**
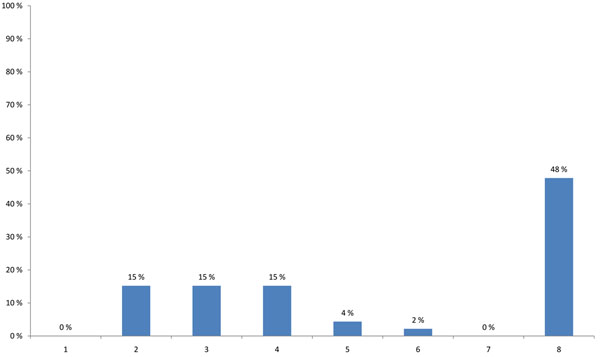
**Stakeholders that the HENVINET portal is most valuable for (multiple choice)** 1 - Scientist at university 2 - Scientist at research institute 3 - Policy-maker at (inter)national level 4 - Policy-maker at regional or local level 5 - Environment and health consultant in private sector 6 - Other relevant social groups such as: Employer organisations, Labour unions, Environmental organisations, Patient groups, Consumer organisations 7 - Citizens 8 - All of the above

**Figure 4:**
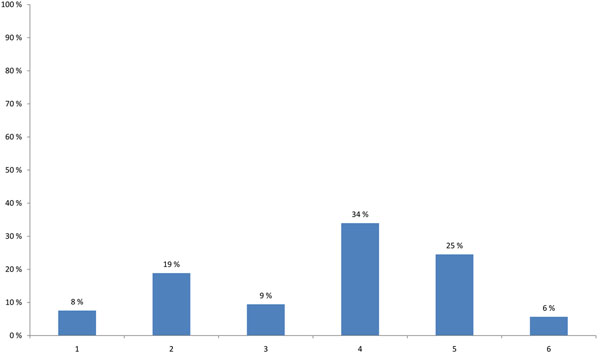
**Actions that could best increase the number of active portal members (multiple choice)** 1 - All HENVINET partners have to contribute with content to the portal. 2 - A paid scientist (task force) should work on building the content of the portal website. 3 - HENVINET should promote the portal with leaflets, email announcements and conference presentations. 4 - The quality of the content of the portal is more important than the amount of information. 5 - The portal should have a lot of additional features such as links to other websites, conference announcements, research calls. 6 - Other actions are better to apply.

HENVINET aimed to increase the number of active portal members with different backgrounds in order to provide diversity to the network communication. 48% of the participants thought that the portal is valuable for a broad range of stakeholders, such as scientists, policy-makers, or consultants; none of the participants believed that the portal was valuable for scientists at universities or the general public (Figure 3). A high percentage of the participants agreed that the quality of the portal content is more important than the amount of information (34%), and that preferably a paid scientist should continuously update the content (19%). One fourth suggested that the portal should have a lot of additional features such as links to other websites, conference announcements or research calls. Only 9% suggested that the best way of promoting the portal would be to use leaflets, e-mail announcements and conference presentations (Figure 4).

**Figure 5:**
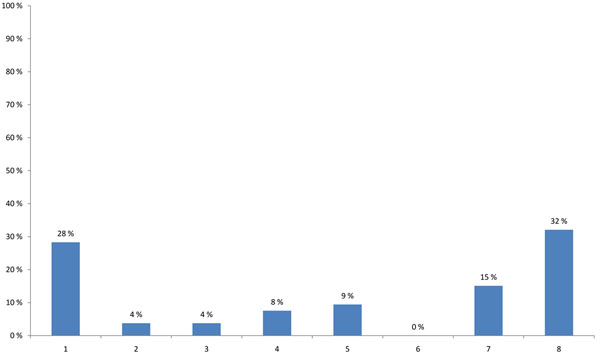
**Most important portal feature to best assist policy-makers in doing their policy work (multiple choice)** 1 - The portal has valuable and scientifically sound information. 2 - The provided information is a confirmation of the information they get from other sources before. 3 - The portal is user friendly. 4 - Make sure that the level of detail is sufficient for their purposes. 5 - The content is only evidence-based. 6 - An automatic system for notifying new messages or items on the portal. 7 - Users can identify on the portal what the current issues are to be considered. 8 - Policy-makers can find experts within their network/field of interest.

**Figure 6:**
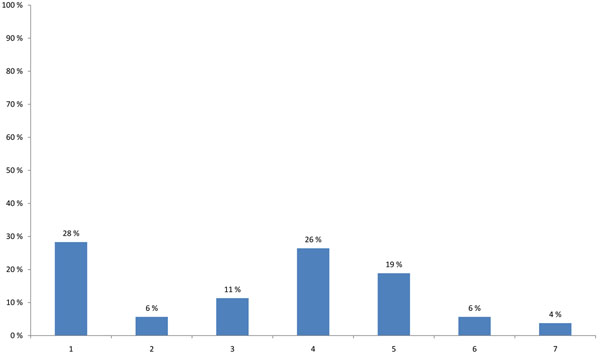
**Most valid statement for making a policy-maker becoming a successful user of the HENVINET portal (multiple choice)** 1 - The policy-maker can interact with a well-known scientist. 2 - The policy-maker can ask a question anonymously to protect his/her own identity. 3 - The policy-maker sees that there is a lot of content on the portal. 4 - The portal provides prepared answers to a range of specific policy issues. 5 - The portal responds within a day to a posed question by a policy-maker. 6 - The portal provides automated lists of topics which are placed on the website. 7 - Other reasons are more valid.

When asked for the portal’s most important feature to assist policy-makers in their work, 32% of the participants assumed that it is up to policy-makers themselves to find scientific experts within their field of interest; they should provide policy-makers with valuable and scientific sound information (28%). 9% declared that the content should only be evidence-based. Another 15% considered the portal’s ability to help the users to identify current issues on E&H as the most important feature (Figure 5). One of the main target groups of the portal users are policy-makers. 28% of the participants regarded the foremost reason for a policy-maker to become a member of the HENVINET portal was the opportunity to interact with well-known scientists directly, and to receive answers to a range of specific policy issues (26%). Another aspect is timeliness: 19% of the participants believed a fast reply by an expert to questions asked on the portal to be the most important reason for policy-makers to become successful users (Figure 6).

### Stakeholder involvement into HENVINET portal activities

**Figure 7:**
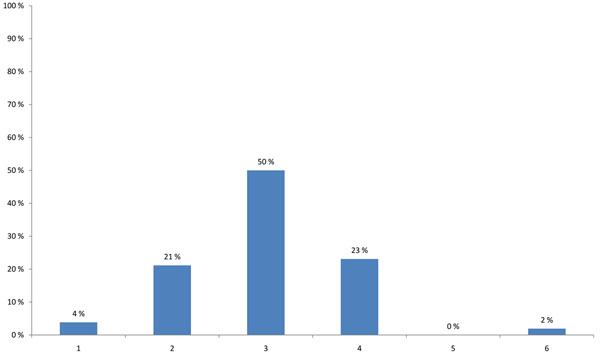
**Willingness to invest time in using a virtual network portal such as the HENVINET portal (multiple choice)** 1 - Yes, to provide content on my own field of interest 2 - Yes, to learn from others and to take information from the portal 3 - Yes, both to provide content and to take information from the portal 4 - Only limited as I get my information mainly from other sources 5 - No, not interested 6 - Only to read the discussions in the discussion groups or forum

Most participants admitted to being beyond their working capacity (‘overloaded’); however, there was no participant who did not show interest in investing time in the use of the HENVINET portal. 50% would be willing both to provide and to take up information from the portal. 23% stated that they would only spend limited time on the portal since they receive their information mainly from other sources and 21% would use the portal to learn from others and take up new information (Figure 7).

### Science-policy interface

**Figure 8:**
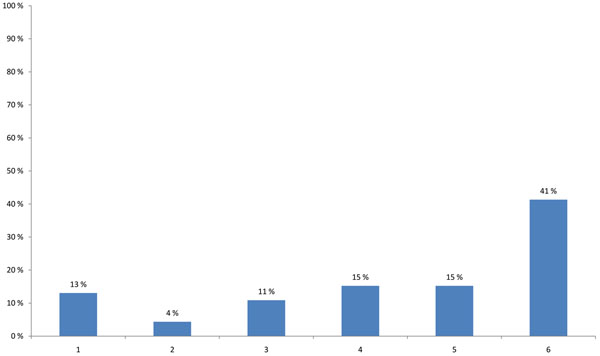
**Most important factor in the development of a policy advise today (multiple choice)** 1 - The policy-maker uses a limited amount of scientists to provide information for a policy advice. 2 - The policy-maker uses a changing group of advisers. 3 - The policy-maker only uses scientific information to support the political opinion of his employer. 4 - The policy-maker wants only evidence-based information to support his/her policies. 5 - Traditional evidence-based culture is in need of critical discussion and innovation because of the limits of current scientific practice with respect to complex important issues in environment and health. 6 - The policy-maker is highly influenced by the media in developing policy statements.

The term science-policy interface is used in this paper to describe any process by which scientific results are taken up by policy-makers. Policy-makers develop their policies based on many kinds of information, ranging from scientific evidence of political and social factors to economics drivers. A large proportion of the participants (41%) had the impression that policy-makers nowadays are highly influenced by the media while developing their policy statements. This influence received higher prioritisation (13%) than the policy-makers’ use of scientific information (11%) (Figure 8).

**Figure 9:**
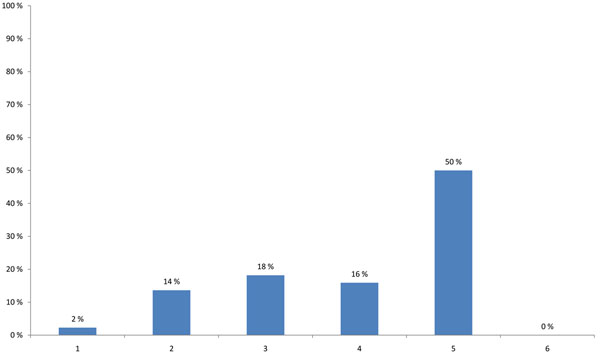
**Most important factor in the development of a policy advise – ideal procedure (multiple choice)** 1 - The policy-maker uses a limited amount of scientists to provide information for a policy advice. 2 - The policy-maker uses a changing group of advisers. 3 - The policy-maker only uses scientific information to support the political opinion of his employer. 4 - The policy-maker wants only evidence-based information to support his/her policies. 5 - Traditional evidence-based culture is in need of critical discussion and innovation because of the limits of current scientific practice with respect to complex important issues in environment and health. 6 - The policy-maker is highly influenced by the media in developing policy statements.

50% of the participants agreed that traditional evidence-based culture is in need of critical discussion and innovation because of the limitations of current scientific practice with respect to complex important issues in E&H. 34% would like to see policy-makers use scientific and evidence-based information to support their policies and the political opinion of their employer, and none of the participants wished to see policy-makers being highly influenced by media (Figure 9).

**Figure 10:**
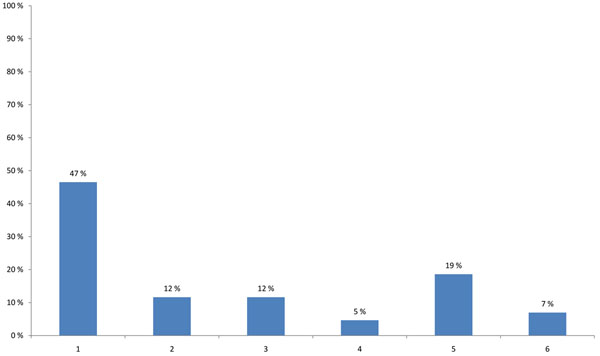
**Best option to make different disciplines work together in tackling environmental health problems (multiple choice)** 1 - The EU has to set up interdisciplinary workgroups on different topics. 2 - The EU has to oblige participants in EU-projects to join the HENVINET portal and add results of their project to the portal. 3 - We have to organise international soccer matches between toxicologists and epidemiologists. 4 - The creation of glossaries (perhaps through a wiki function) for scientific terms and policy terms. 5 - Continuous professional education needs to include obligatory courses of other disciplines. 6 - I have a great idea myself.

In order to make different disciplines work together in tackling environmental health problems, almost half of the participants (47%) agreed that the EU has to set up interdisciplinary workgroups on different topics. This statement was supported by 19% saying that continuous professional education also needs to include obligatory courses in other disciplines to encourage multi-disciplinary approaches (Figure 10).

### Decision support tools (DSTs)

**Figure 11:**
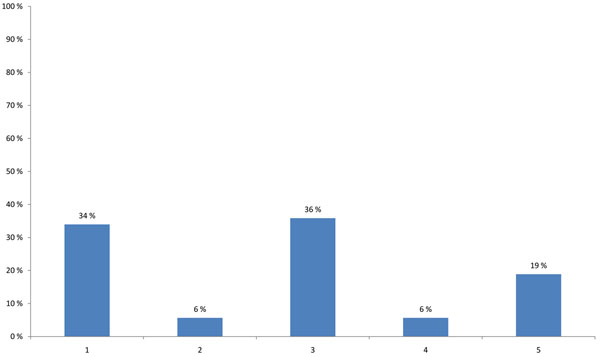
**Most important factor in the use of decision support tools (DSTs) (multiple choice)** 1 - HENVINET should play a role in distribution on the possible use of decision support tools. 2 - Policy-makers have enough insight in the use of decision support tools. 3 - Decision support tools can only be used when they have been sufficiently validated. 4 - Decision support tools are overrated instruments. 5 - Researchers have to use decision support tools and give the results to policy-makers.

HENVINET produced a searchable Meta Data Base (MDB) of E&H decision support tools (DSTs) to support policy-makers in their decision-making process. 34% of the participants would like to see HENVINET playing a role in distributing information about the use of DSTs whereas 6% considered DSTs to be overrated instruments. One third of the participants (36%) thought that DSTs can only be used when they have been sufficiently validated. The participants also agreed that there is a lack of knowledge regarding the proper use of DSTs by policy-makers: only 6% of the participants believed that policy-makers have enough insight in the use of DSTs and 19% suggested researchers should use DSTs but give only the results to policy-makers (Figure 11).

## Discussion

The results of the HENVINET voting session have been used as feedback to evaluate the social media approach of the HENVINET networking portal to communicate E&H issues. One should bear in mind that the voting session had some limitations: the number of participants (53) who took part in the survey is limited, and about half of the attendees already had gathered some experience in working with the portal due to the fact that they were a part of the HENVINET consortium. Moreover, as mentioned earlier, participants only had one vote – and giving priority to one answer does not necessarily mean the other issues are being regarded as unimportant. There might also be some information bias, based on fresh information from presentations held in previous sessions during the conference.

### Stakeholder analysis

The HENVINET portal was created as a platform to bring together stakeholders from different backgrounds to bridge the gap between science and policy in the complex fields of E&H. Uncertainty is one of the main problems decision-makers have to deal with when assessing, managing and communicating risks of environmental effects on human health [8]. As outcomes from previous projects (e.g. AIRNET) indicate, close cooperation between stakeholders with different professional backgrounds is essential for more effective management of uncertainties and development of widely supported policies [19]. The project consortium therefore invited EU stakeholders with different backgrounds to join the final project event in Brussels: EC commissioners, members of EEA and WHO, participants from other comparable projects and private networks. Additionally, each member of the HENVINET portal (ca 300 at the point of the voting sessions) received an invitation. However, the majority of the participants attending the voting session had a scientific background, which leads to a biased presentation of results, reflecting to a greater extent the scientists’ viewpoint.

The participants agreed that the HENVINET portal is suitable for a broad number of stakeholders – ranging from scientists and policy-makers to E&H consultants and other relevant social groups (patient groups, consumer organisations, etc). Nevertheless, two exceptions were named: scientists at universities and the general public. For the general public the information obtained in the HENVINET portal might be too scientific whereas scientists in the academic environment seem to use different modes of receiving and forwarding information, applying their own language and priorities [20]. One has to take into consideration that policy-makers are not one homogenous group but come from different backgrounds; some policy-makers for example can be considered to be experts as well as scientists. The results of the voting session, however, do not allow conclusions to be drawn about the background of the participating policy-makers.

### Stakeholders’ needs

In order to raise the number of active portal members and best support *‘better informed policy making’ *[15], the participants of the voting session put strong emphasis on the quality of the portal content. The vast majority set the improvement of the quality of the portal content as priority, e.g. by hiring a dedicated scientist to work on improving content quality. Particular attention should be paid to scientifically sound information, best in combination with direct contact with an expert in the policy-makers’ field of interest. As *Clark & Holmes* report, decision-makers often do not know whom to contact for (specific) scientific information. To get more opportunities to establish personal contact with scientists in addition to conferences and meetings, they wish for an electronic searchable database of experts whom they can approach directly on specific issues [20]. Several other study findings also report that decision-makers put strong emphasis on the importance of using the internet and suggest a one-to-one interaction with researchers to discuss research findings and potential implications for practice [13,21]. Also a systematic review by *Innvær* et al. clearly demonstrates the importance of personal contact between researchers and policy-makers to facilitate the use of research evidence in policy making [22]. To become a successful user of the HENVINET portal, policy-makers should therefore have the possibility to expand their personal network by interacting directly with established scientists and be provided with answers to a range of specific policy issues. This would not only serve decision-makers, but also scientists in turn would benefit from this dialogue by getting more insight into precisely what research is policy relevant. The possibility to address an expert in one’s own field of interest at the HENVINET portal is already available. The search function allows registered portal users to search members by country, interest area, etc. and contact them directly.

In this context, portal members providing scientific information for policy-makers should pay special attention to the format of their contribution. In order to assure effective access to and uptake of information and expertise, study outcomes indicate that research results should be readily available, most suitably in the form of a searchable database or single web entry point [20,23]. There are different formats that can be chosen to deliver scientific results, depending on the target audience: reports, peer reviewed articles, policy briefs, etc., to mention only a few. To ensure that the information is effectively disseminated, one should bear in mind that policy and decision-makers suffer from a lack of time and a flood of information; scientific information therefore should be short, easy to digest and understandable for lay people [24]. There are policy-makers using peer reviewed articles as a source of information. However, due to time constraints and lack of direct access, journal articles are not necessarily the first choice as dissemination material to be used within decision-making processes. More useful instruments are reports including a summary of key findings in combination with a reliable description of context and policy implications to make them applicable to specific national or international contexts [23,25]. Also, not every policy-maker has a scientific background and is able to comprehend scientific reports. For this purpose the HENVINET project has been developing a series of policy briefs that are available at the HENVINET portal. They are the results of web-based expert knowledge evaluations (see [26-30]) and expert elicitation workshops [31], giving a short overview of a scientific problem and offering specific options to take up scientific results into the development of concrete policy actions. Each brief consists of policy context, policy options, an executive summary and recommendations with references, allowing the user to trace the information that is used for the brief (available at http://www.henvinet.eu). The beneficial use of policy briefs to communicate research results to policy-makers has also been reported by *Jones & Walsh*. Their findings underline that policy briefs are valuable communication tools for dissemination of research results [25]. Currently the creation of policy summaries is oftentimes not regarded as part of a scientist’s job, due to a lack of necessary communication skills. Most scientists are not trained in communication and/or do not have direct contact with decision-makers: for this reason they have difficulties finding out what research is actually policy relevant and seeing the necessity of policy briefs [19,20]. Therefore the design of policy briefs and subsequent ‘translation’ of scientific results turn this context-relevant guidance into a ‘language’ that is understandable for policy-makers; this work can be carried out by an intermediary – a specially trained expert or communication specialist. Particularly in the complex field of environmental health it is crucial to carry out a successful communication process between different stakeholders [32]. Intermediaries should preferably have a background in natural or social sciences, good communication and inter-personal skills, experience in policy work, awareness of ‘the bigger picture’, and a good judgement to guarantee an active mediation and translation process [23,25]. Such an expert could be hired for working on the content of the HENVINET portal to ensure its high quality standard, which was also a key suggestion at the voting session.

To attract new members, the provision of additional features in the HENVINET portal, such as links to other websites, research calls, etc., seemed to be of great importance to the participants of the voting session. Issues like user friendliness or level of detail, however, appear to be less important for the participants, an indication that the portal is either already considered user friendly or that the criteria ‘user friendliness’ is less important in comparison to the other issues named in Figure 5.

### Science-policy interface

The HENVINET portal was designed to support policy-makers in their decision-making process by providing adequate information, ‘fit for purpose’. The majority of the participants believe that policy-makers are generally highly influenced by media in the process of information collection and decision-making. It was further claimed that in order to develop information that is most suitable for policy-makers there is a strong need for alternatives to the traditional evidence-based research; however, there were no specific suggestions made how these alternatives could/should look.

Interdisciplinary approaches are considered as important pre-conditions for the work in E&H. 47% of the participants suggested the EU should set up interdisciplinary workgroups on different topics to work together in tackling E&H problems (Figure 10). A similar approach has been chosen by the EU project AIRNET. The close cooperation of a broad range of stakeholders and different scientific disciplines has led to a more effective management of uncertainties and the development of widely supported policies [19]. Similar advantages have been reported from the ‘Sandpit’ approach of the UK Engineering and Physical Sciences Research Council, an interactive multidisciplinary workshop bringing together a number of different stakeholders to address different research challenges [33]. Interdisciplinarity is also a key principle for the HENVINET portal, and close cooperation between different stakeholders and scientists from different backgrounds marks a core element in the support of decision-makers.

### Decision support tools (DSTs)

Besides developing concrete policy advice, the research community can also assist policy-makers by providing decision support tools (DSTs). This term describes a range of instruments that can be applied by policy-makers to give them an improved basis for their decision-making process. One of the models that has been developed by the HENVINET project, was a number of causal chain diagrams. The E&H issues that have been identified within the project were translated into a schematic framework which was illustrated by a causal chain diagram to identify and illustrate the links between environmental change and its consequences on health. Based on the diagram, a web-based questionnaire was developed to ask experts to assess the diagrams’ completeness and accuracy, and the state of knowledge in each element and associated link. As a final step, the experts analysed and interpreted agreements and disagreements in their answers and, based on the results, suggested prioritised actions which were translated into policy briefs. Seven diagrams on the following topics are currently available for expert evaluation through the HENVINET web site: Asthma and Allergy, Chlorpyrifos, Cancer, Phthalates, BFR (decaBDE, HBCD), (see also [26-30]) and Nanoparticles. The HENVINET project has also prepared a searchable database of different DSTs in the field of E&H that is accessible via http://www.henvinet.eu. According to the results of the voting session, the HENVINET project should actively distribute these DSTs. However, the tools should only be used after they have been sufficiently validated. The voting participants do not seem to trust the policy-makers’ insight in applying them in the way they were meant to be used. Instead, it was suggested that policy people should make use of their scientific contacts within the portal and let them use the tools upon request and forward only the results back to the policy-makers.

### Social media approach as mode of two-way-communication in E&H

The main objective of the HENVINET project was to establish a long-term co-operation between researchers and policy-makers (and to a certain degree other stakeholders) in the area of E&H. To reach this aim, effective two-way communication is an indispensable tool. Communicating E&H issues is not only restricted to the dissemination of scientific results to policy-makers, but also the other way round – using decision-makers’ (prioritized) experience to shape and develop scientific research. Outcomes from the AIRNET project for example show that a close cooperation between different stakeholders leads to a *‘common understanding and respect for each other’s challenges and dilemmas’*. This can result in better framing of research programs that are *‘more targeted to what the stakeholders need and what the scientists consider possible’ *[19]. As *Clark & Holmes* report, scientists often do not know what kind of research is policy relevant due to a lack of communication and knowledge about whom to contact. It was suggested that *‘improved interface between the different social structures and increase shared understanding of research and policy work’* should be provided by more opportunities and through better ways for policy people, researchers, experts and further stakeholders to get into and maintain these contacts [20]. This will also lead to an improved understanding for scientists of what information policy people may regard as ‘*timely, relevant or good quality research*’ [22].

## Conclusions

With regard to these study findings, more emphasis should be put on a balanced two-way-communication for future activities at the science-policy interface. The one-to-one interaction the HENVINET portal offers (as it has been described above) is therefore a very useful function to improve interaction between science and policy or further stakeholders. Information can flow into both directions, from policy to science and from science to policy to set priorities in the research agenda and shape the development of policies.

Policy-makers currently employ various sources to acquire necessary information for policy decisions, with the internet being the main source; social media provide new approaches for the dissemination and communication process in this context. The HENVINET portal offers clear advantages; it is interactive and simple to use, it supports individual as well as group communication, and it provides access to tools for decision-making – elements that facilitate communicating in the area of E&H. In this way, the portal brings the communication process necessary to make scientifically sound decisions and policies nearer to the sought-after dialogue-based ideal. In this context, sustained resources are needed to develop HENVINET portal content and to encourage participation.

The work towards creating long-term collaboration between stakeholders in the E&H field has been advanced through the activities described in this article. Improved decision-making requires active participation by the policy side within the dialogue with science, but no empirical evidence is available yet to what extent the portal has contributed here.

## Competing interests

The authors declare that they have no competing interests.

## Authors' contributions

All authors planned this work. SG wrote the manuscript, PvdH and AB made the first revision and gave valuable input. All authors approved the final version.

## References

[B1] Duncan D Clickers in the Classroom: How to Enhance Science Teaching Using Classroom Response Systems 2005 Addison-Wesley

[B2] Lundgren RE Risk communication: a handbook for communicating environmental, safety, and health risks 2009 4 Columbus Battelle Press

[B3] O'Fallon LR Dearry A Community-based participatory research as a tool to advance environmental health sciences Environ Health Perspect 2002 110 Suppl 2 155 159 11929724 10.1289/ehp.02110s2155 PMC1241159

[B4] Nowotny H Scott P Gibbons M Introduction: ‘Mode 2’ Revisited: the New Production of Knowledge Minerva 2003 41 179 194 10.1023/A:1025505528250

[B5] Janssen PHM Petersen AC van der Sluijs JP Risbey JS Ravetz JR A guidance for assessing and communicating uncertainties Water Science & Technology 2005 52 125 131 16304944

[B6] Ravetz J Funtowicz S Post-Normal Science-an insight now maturing Futures 1999 B31B 641 646

[B7] Dobbins M Ciliska D Cockerill R Barnsley J DiCenso A A Framework for the Dissemination and Utilization of Research for Health-Care Policy and Practice The Online Journal of Knowledge Synthesis for Nursing 2002 9 Document No7 12439759

[B8] Mossman KL Policy decision-making under scientific uncertainty: radiological risk assessment and role of expert advisory groups Health Phys 2009 97 101 106 10.1097/HP.0b013e3181a7abf2 19590269

[B9] Klein R Evidence and policy: interpreting the Delphi oracle J R Soc Med 2003 96 429 431 10.1258/jrsm.96.9.429 12949196 PMC539595

[B10] Engels A The Science-Policy Interface IAJ 2005 5 7 26

[B11] Social Media http://en.wikipedia.org/wiki/Social_media

[B12] Kaplan AM Haenlein M Users of the world, unite! The challenges and opportunities of Social Media Business Horizons 2010 53 59 68 10.1016/j.bushor.2009.09.003

[B13] Dobbins M Jack S Thomas H Kothari A Public Health Decision-Makers’ Informational Needs and Preferences for Receiving Research Evidence Worldviews on Evidence-Based Nursing 2007 4 156 163 10.1111/j.1741-6787.2007.00089.x 17850496

[B14] EU Communication from the Commission to the Council the European Parliament the European Economic and Social Committee “The European Environment & Health Action Plan 2004-2010” COM, 416 final 2004

[B15] Randall S Yang A Kobernus M Bartonova A HENVINET technical tools (Final report for Work Package 2) 2010 NILU OR 69/2010

[B16] Google Analytics Dashboard Record for www.henvinet.eu, 01 July 2009-28 February 2011 https://www.google.com/analytics/reporting/?reset=1&id=24550463&pdr=20110201-20110303

[B17] Elliott C Using a Personal Response System in Economics Teaching International Review of Economics Education 2003 1 80 86

[B18] Roschelle J Penuel WR Abrahamson L Classroom Response and Communication Systems: Research Review and Theory Paper presented at the Annual Meeting of the American Educational Research Association, San Diego, CA, April 2004

[B19] Totlandsdal AI Fudge N Sanderson EG van Bree L Brunekreef B Strengthening the science-policy interface: experiences from a European Thematic Network on Air Pollution and Health (AIRNET) Environmental Science & Policy 2007 10 260 266 10.1016/j.envsci.2007.01.003

[B20] Clark R Holmes J Improving input from research to environmental policy: challenges of structure and culture Science and Public Policy 2010 37 751 764 10.3152/030234210X534887

[B21] Pineault R Tousignant P Roberge D Involving Decision-Makers in Producing Research Syntheses: The Case of the Research Collective on Primary Healthcare in Quebec Healthcare Policy 2007 2 e193 209 19305728 PMC2585463

[B22] Innvær S Vist G Trommald M Oxman A Health policy-makers’ perceptions of their use of evidence: a systematic review J Health Serv Res Policy 2002 7 239 244 10.1258/135581902320432778 12425783

[B23] Holmes J Clark R Enhancing the use of science in environmental policy-making and regulation Environmental Science & Policy 2008 11 702 711 10.1016/j.envsci.2008.08.004

[B24] Sorian R Baugh T Power Of Information: Closing The Gap Between Research And Policy Health Affairs 2002 21 264 273 11900168 10.1377/hlthaff.21.2.264

[B25] Jones N Walsh C Policy briefs as a communication tool for development research ODI Background Notes 2008

[B26] Forsberg B Bråbäck L Keune H Kobernus H Krayer von Krauss M Yang A Bartonova A An expert assessment on climate change and health – With European focus on lungs and allergies Environmental Health 2012 11 Suppl 1 S4 10.1186/1476-069X-11-S1-S4 PMC3388443 22759504

[B27] Saunders M Magnanti BL Correia Carreira S Yang A Alamo-Hernández U Riojas-Rodriguez H Calamandrei G Koppe JG Krayer von Krauss M Keune H Bartonova A Chlorpyrifos and neurodevelopmental effects: A literature review and expert elicitation on research and policy Environmental Health 2012 11 Suppl 1 S5 10.1186/1476-069X-11-S1-S5 PMC3388448 22759505

[B28] Merlo DF Filiberti R Kobernus M Bartonova A Gamulin M Ferencic Z Dusinska M Fucic A Development of causal diagrams for cancer Environmental Health 2012 11 Suppl 1 S9 10.1186/1476-069X-11-S1-S9 PMC3388474 22759509

[B29] Zimmer KE Gutleb AC Ravnum S Krayer von Krauss M Murk AJ Ropstad E Skaare JU Sundstøl Eriksen G Lyche JL Koppe JG Magnanti BL Yang A Bartonova A Keune H Policy relevant results from an expert elicitation on the health risks of phthalates Environmental Health 2012 11 Suppl 1 S6 10.1186/1476-069X-11-S1-S6 PMC3388473 22759506

[B30] Ravnum S Zimmer KE Keune H Gutleb AC Murk AJ Koppe JG Magnanti BL Lyche JL Eriksen GS Ropstad E Skaare JU Kobernus M Yang A Bartonova A Krayer von Krauss M Policy relevant Results from an Expert Elicitation on the Human Health Risks of Decabromo-diphenyl ether (decaBDE) and Hexabromocyclododecane (HBCD) Environmental Health 2012 11 Suppl 1 S7 10.1186/1476-069X-11-S1-S7 PMC3388476 22759507

[B31] Keune H Gutleb AC Zimmer KE Ravnum S Yang A Bartonova A Krayer von Krauss M Ropstad E Eriksen GS Saunders M Forsberg B Fucic A We’re only in it for the knowledge? A problem solving turn in environment and health expert elicitation Environmental Health 2012 11 Suppl 1 S3 10.1186/1476-069X-11-S1-S3 PMC3388440 22759503

[B32] Beato RR Telfer J Communication as an Essential Component of Environmental Health Science Journal of Environmental Health 2010 20687329

[B33] EPSRC Sandpits http://www.epsrc.ac.uk/funding/grants/network/ideas/Pages/whatisasandpit.aspx

